# The Energetic Cost of Walking: A Comparison of Predictive Methods

**DOI:** 10.1371/journal.pone.0021290

**Published:** 2011-06-22

**Authors:** Patricia Ann Kramer, Adam D. Sylvester

**Affiliations:** 1 Departments of Anthropology and Orthopaedics and Sports Medicine, University of Washington, Seattle, Washington, United States of America; 2 Max Planck Institute for Evolutionary Anthropology, Leipzig, Germany; Universidad Europea de Madrid, Spain

## Abstract

**Background:**

The energy that animals devote to locomotion has been of intense interest to biologists for decades and two basic methodologies have emerged to predict locomotor energy expenditure: those based on metabolic and those based on mechanical energy. Metabolic energy approaches share the perspective that prediction of locomotor energy expenditure should be based on statistically significant proxies of metabolic function, while mechanical energy approaches, which derive from many different perspectives, focus on quantifying the energy of movement. Some controversy exists as to which mechanical perspective is “best”, but from first principles all mechanical methods should be equivalent if the inputs to the simulation are of similar quality. Our goals in this paper are 1) to establish the degree to which the various methods of calculating mechanical energy are correlated, and 2) to investigate to what degree the prediction methods explain the variation in energy expenditure.

**Methodology/Principal Findings:**

We use modern humans as the model organism in this experiment because their data are readily attainable, but the methodology is appropriate for use in other species. Volumetric oxygen consumption and kinematic and kinetic data were collected on 8 adults while walking at their self-selected slow, normal and fast velocities. Using hierarchical statistical modeling via ordinary least squares and maximum likelihood techniques, the predictive ability of several metabolic and mechanical approaches were assessed. We found that all approaches are correlated and that the mechanical approaches explain similar amounts of the variation in metabolic energy expenditure. Most methods predict the variation within an individual well, but are poor at accounting for variation between individuals.

**Conclusion:**

Our results indicate that the choice of predictive method is dependent on the question(s) of interest and the data available for use as inputs. Although we used modern humans as our model organism, these results can be extended to other species.

## Introduction

Determining the amount of energy that animals devote to movement in their environment has been an area of intense interest to biologists for decades (e.g. [1]) and much research effort has been devoted to teasing apart the many and varied possible causative agents. The principal reason for this scrutiny is that energy is a finite, non-recyclable resource [2]—energy used to move is lost to reproduction, an activity that is both energetically intensive for mothers (e.g. [3]) and sensitive to energetic restriction as reflected in maternal body mass (e.g. [4], [5], [6], [7], [8], [9], [10]). Consequently, those animals that use less locomotor energy to accomplish their activities of daily living should leave more offspring [11].

Understanding energetic expenditure in the body is complicated, because the body is a machine that is capable of performing a complex conversion of energy [12]. Chemical energy enters the body in the form of nutrients (usually obtained through ingestion) and oxygen (through respiration) and is used by the body to fuel internal chemical processes, to move (mechanical energy), and to create heat (thermal energy) [12]. The system is not 100% efficient and some wastage occurs [13]. Energy can also be stored over the short-term (e.g. elastic energy in tendons) or long-term (e.g. fat mass). Although the fundamentals of this transformation are understood (e.g. the conservation of energy dictates that energy input must equal energy output plus storage), the manner in which energy is distributed among the muscles and other tissues to produce stability and movement are not currently fully understood [14] or capable of being monitored [15], i.e., the system remains something of a “black box.” Nonetheless, both the amount of tissue activated (muscle volume) and the intensity of the movement seem to affect the amount of metabolic energy used and over the decades of research, two basic methods to understand locomotor energy expenditure have emerged: those based on metabolic energy and those based on mechanical energy.

### Metabolic energy

Metabolic approaches are the most direct of the two methods because they predict metabolic function from proxies of it, such as the body's demand for ATP. The most commonly used proxy of metabolic function is the volumetric rate of oxygen consumption (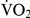), which serves as an estimate for ongoing cellular respiration and thus the body's use of energy; we will employ this proxy here. At this time, metabolic energy consumption approaches to the study of locomotor energy expenditure are inherently empirical and they employ statistical techniques to predict the dependent variable oxygen consumption from independent covariates (e.g. [16], [17], [18], [19], [20]). The classic papers of Taylor and his many colleagues (e.g. [21], [22], [23], [24], [25]) established that the independent variables velocity (a proxy for movement intensity) and body mass (a proxy for muscle volume) are predictive of oxygen consumption across a wide range of animal orders [13], [26].

**Figure 1:**
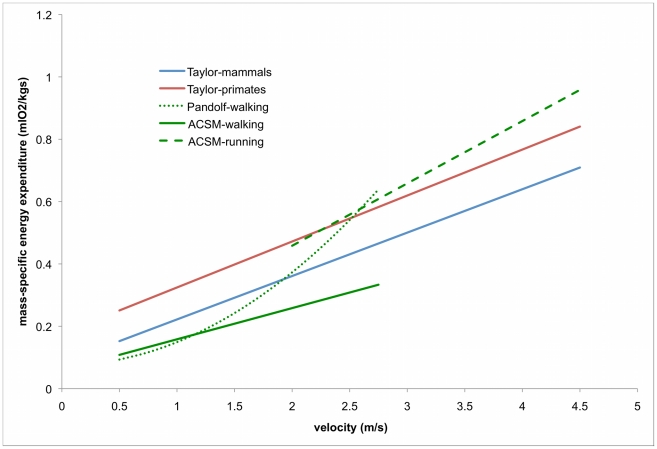
Relationship between velocity and locomotor energy expenditure. The equations for mammals (blue line) and primates (red line) are from Taylor and colleagues (1982); the ACSM equations for human walking (green line) and running (green dashed line) are from the ACSM handbook [20]; the curvilinear human walking equation (green dotted line) is from Pandolf and colleagues [16]. All calculations assumed a body mass of 70 kg.

**Figure 2:**
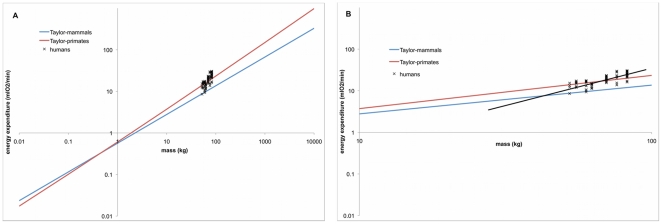
Relationship between body mass and locomotor energy expenditure. A) “Mouse-to-elephant” scale for body mass. B) Human scale for body mass. Equation for mammals (blue line) and primates (red line) from Taylor and colleagues (1982). Individual human data from the current study. Linear regression line through human data (black line) provided fro reference only.

The independent covariates of “mouse-to-elephant” relationships have also been shown to be predictive of oxygen consumption over groups with less variability (e.g. within a species), but the predictive relationships are different (e.g. [16], [19], [27]). For instance, Figure 1 describes the general mammalian, primate, and human patterns that relate velocity and the volumetric rate of oxygen consumption per kg of body mass (from [16], [20], [24]). As is apparent, as velocity increases, energy expenditure per kg body mass increases, but how fast that increase occurs depends on the group and, for humans at least, the gait of interest. Consequently, while it is true to say that velocity is a significant predictor of energy expenditure in all these groups, knowing the relationship across primate genera does not necessarily lead to accurate predictions within a single species like *Homo sapiens*. The same is true for body mass—body mass is a statistically significant predictor of energy expenditure intra- (within *Homo sapiens*) and inter-specifically (within *Primates*), but the intra-specific relationships are different from the inter-specific ones (Figure 2) [17].

Consequently, a predictive equation for energy expenditure, developed from the data of one species, cannot be assumed to be accurate to predict the energy expenditure of another. The same is true in reverse: predictive equations developed from multiple species may lead to inaccuracies in prediction within single species. This same phenomena may also be at work at scales other than species, such as populations or groups (e.g. the differences in basal or resting metabolic rates between populations living in cold and hot climates [28]). Simply extrapolating equations developed from empirical studies of one group can, therefore, lead to wrong results in another, necessitating that metabolic approaches be grounded by a theoretical underpinning that delineates how the differences in morphology and physiology affect energy expenditure.

### Mechanical energy

If all groups of interest were amenable to direct metabolic testing, mechanical approaches would be unnecessary. Such is not the case, however, so mechanical energy approaches are important because they may offer some insight into how energy expenditure changes with morphology and gait characteristics and can help explain why equations are not universally applicable across and between species and populations.

The mechanical approaches have focused on the energy output side of the equation (specifically, the energy of movement). All mechanical energy solutions utilize first principles, namely, the Newtonian laws of motion and conservation of energy, to calculate mechanical energy expenditure. These calculations can, therefore, be completely theoretical. Most researchers, however, employ theoretical constructs that are tested empirically, often using statistics. Mechanical energy approaches can address the problem of predicting locomotor energy expenditure from many different angles. This fundamental difference between metabolic and mechanical approaches makes mechanical approaches attractive to study extinct creatures and those difficult to study in the laboratory [29], [30], [31], [32], even though mechanical approaches cannot represent non-mechanical phenomena (e.g. efficiency differences in cardiovascular function among individuals).

#### Energy of motion

One approach to predict the energy expenditure of walking is to assess the change in the body's potential and kinetic energy. Margaria, Cavagna and colleagues were among the first researchers to combine body mass with the movement of the body's center of mass to assess energy expended, or work done [33]. The movement of the body's center of mass was determined either using a recording of the movement of a marker placed near the center of mass [34] or from the force that the body exerted against the ground [33], i.e., the ground reaction force. These methods can be shown to be equivalent [35] and their choice depends on the availability of equipment to record the motion or the force. Both of these methods address the effect of the amount of tissue activated and intensity of motion.

A second approach is to model the lower body as rigid links that move together to produce the motion of the whole body. In inverse dynamic solutions, the model is driven by motions, while in forward dynamic solutions, external forces are used. Inverse and forward dynamic models have been used to calculate the energy required to move the limbs (dubbed internal work while that of the whole body is designated external work) at the same time as the external work either by using joint moments [36] or by combining segmental energy changes with calculated external work [37]. It is also possible to use a looping process where inverse dynamics is informed by forward dynamics [38].

#### Muscular energy

Another approach that uses mechanics to predict metabolic function has focused on the production of muscular force using several different methods. One method, force production which was originally developed for running [39], but later extended to walking [40], begins with the hypothesis that the primary cost of locomotion is the cost of generating the muscular force necessary to move the animal. These approaches are theoretically similar to center of mass motion or ground reaction force approaches. Several assumptions are used to operationalize the muscular force production methods: 1) that most of the force generated by the muscles is used to oppose gravity, 2) that a volume of muscle exerts the same force against the ground regardless of an animal's size, shape or speed, and 3) that muscles operate over the same range of the force-velocity curve [39]. With these assumptions in place, the authors develop an equation that makes energy expenditure proportional to body weight divided by contact time ([39], equation 1, p. 265). This method, with the simplifying assumptions in place, becomes a restatement of the empirical result that mass ( =  weight/gravitational constant) and velocity (as measured by time) are predictive of locomotor energy expenditure.

A second method used to predict muscular force is to model muscles as elements in a multi-segment dynamic model. Dynamic (usually forward dynamic) models are used to create/predict muscle activation sequences that can then be used to calculate muscular energy usage [32], [41], [42], [43]. Another method that uses activated muscle volume (as a proxy of muscular energy use) to predict metabolic energy has recently become available [44], but the technique is applicable only at the species-level because it relies on muscle volume from dissection.

Approaches that emphasize the prediction of muscular forces and energy are different from those that utilize segment and/or whole body potential and kinetic energy, because they are based on creating activation in muscles which can be translated to metabolic energy use. Given that muscular processes are intermediate steps between the intake of nutrients and movement in the environment, methods to predict the force in specific muscles are exciting advances in the field of biomechanics, with the ability to predict 85-90% of the variability in the energy expenditure of walking [45]. In addition to probing deeper into the “black box” of whole-body energy conversion, they have the potential to address the energy expended by non-motion, e.g. joint stabilization via co-contraction. Given the complexity of the calculations that underlie these approaches, a software platform is necessary to organize the input and output data and several commercial and public systems are under-development. The implementation of the software is, however, still incomplete. For instance, co-contraction is not yet possible [42] and it not currently possible to model the potential differences in muscle activation among individuals seen in recent electromyography studies (e.g [46], [47]). When muscle force models can be tailored to individuals, we look forward to extending this work to these models.

#### Generalizations about mechanical approaches

All the mechanical calculations are based on the same theoretical underpinnings and consequently, the same limitations apply to all: mechanical calculations require knowledge of the motions or forces involved and the physical characteristics of the system (e.g. mass and lengths). Table 1 indicates the critical equations and variables needed to make the calculations using the various schemes. The upshot is that all of these methods require assumptions about the characteristics of the system that are needed as inputs in order to calculate energy and some variables are easier to measure than others. For instance, total body mass can be measured with a readily available instrument that has low error. The movement of an anatomical landmark, however, requires sophisticated equipment that has higher error. Some variables, like mass moment of inertia, are impossible to measure in living creatures and have to be estimated. Because any mechanical simulation of reality is only as good as the assumptions and simplifications that go into running it, the quality of the inputs are a critical component in the choice of methodology. The underlying theory can be correct, but produce seemingly incorrect results simply because the system was not modeled accurately. The choice among the methods is, then, made by evaluating which method requires the type (including accuracy) of inputs one has available and provides the output data to answer the questions in which one is interested.

**Table 1:** Predictive methods, input variables and critical equations.

Method	Variable name	Methodology	Input variables	Critical equations
ACSM-walk	ACSM	Statistical	Velocity (v), body mass (m), regression coefficients	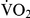 = (0.1v+0.0583)m
Force production	EXT-FP	Measured, calculated, statistical	Body mass, velocity, ground contact time	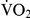 = ƒ (vm/contact time)
CoM-GRF	EXT-GRF	Measured, calculated	Ground reaction forces, body mass, acceleration (a)	F = mav = ∫a dtEnergy = mgh+0.5mv^2^External work = ∑ dEnergy (time)
CoM-sacrum-model	EXT-MAT	Simulated	Velocity, body mass, segment lengths, angles	**x** = ƒ (segment length, angles)v = d**x**/dtEnergy = mgh+0.5mv^2^External work = ∑ dEnergy (time)
CoM- sacrum-measured	EXT-SAC	Measured, calculated	Motion of sacrum (**x**), body mass	v = d**x**/dtEnergy = mgh+0.5mv^2^External work = ∑ dMEnergy (time)
Internal work	INT-MAT	Simulated	Velocity, angular velocity (ω), segment lengths, angles, segment mass and moments of inertia (I)	Energy = ∑ 0.5 (mv^2^ + Iω^2^)(of segments)Internal work = ∑ dEnergy (time)
Joint moments	COMB-JM	Measured, calculated	Ground reaction forces (F), joint shape (r/R), motion of ankle, knee and hip joints	Muscle force = r/R FPower = Muscle force * vCombined work = ∑ Power/time (of joints)
Model (int + ext work)	COMB-MAT	Simulated, calculated	Internal (INT-MAT) and external (EXT-MAT) work	Combined work = internal +external work

Note that the ACSM method is a metabolic energy approach while others are mechanical energy approaches. The mechanical energy approaches are grouped into those the approximate external work (the energy required to move the body), internal work (the energy required to move the legs relative to the body) and combined work (external and internal work).

We should note that although modern humans and their ancestors have received the lion's share of attention and most of the examples used herein are drawn from that arena, the methods we discuss are as appropriate for use in other species. For instance, the locomotion of gibbons [48], Japanese macaques [49], [50], horses [51], [52], cockroaches [53], salamanders and tuataras [54], and extinct non-avian dinosaurs [55] have been evaluated using some of the general methods discussed here. Modern humans are not special, but merely convenient, organisms in which to study the energetics of locomotion.

### Summary

When the creatures of interest are suitable for direct observation and study, the metabolic approach seems appropriate. Unfortunately, because no general statistical formula has been establish (i.e., there are different equations for different situations), equations are only valid for the group from which they were developed. If these group-appropriate equations are developed, then direct calculation of actual energy requirements might be obtainable. This seems to be particularly important for studies where the goal is to assess the absolute total daily energy expenditure for a group as in situations where caloric supplementation (due to high energy requirements and low reserves) or restriction (due to high reserves or low energy requirements) is necessary.

When the creatures cannot be directly studied, as is the case with extinct creatures, the best that can be done is to rely on the theoretically-based mechanical energy approaches, where one can vary the assumptions, variables of interest, and/or methodology. Actual, or absolute levels of, energy expenditure cannot be assessed, but it should be possible to compare relative values. This allows investigation of the effect of different morphologies or to assess the effect of one variable on another. Examples of this might be to compare the effect of different crural indices or of segment length and circumference combinations on the cost of locomotion in modern humans and neandertals, in which direct experimentation is impossible [56], [57]. Mechanical models also offer the ability to explore hypothetical morphologies. An example of this scenario would be the suggestion by Lovejoy and colleagues [58], [59], [60] that hominin bipedalism evolved from a monkey-like quadrupedal ancestor rather than from a knuckle-walking ape-like ancestor.

A logical initial step, however, is to assess the effect of choice of methodology on the prediction of energy expenditure. In order to do this, the detailed morphological, metabolic, kinematic and kinetic data of individuals are needed. The inherent assumption here is that the variability expressed by individuals within a species is sufficient to represent the effect of methodological choice across species. Modern human adults were chosen for the study detailed herein, because as noted above they are readily available as test subjects, they are generally compliant with the testing conditions, and their metabolic and mechanical energy expenditure has been extensively studied. Our techniques and findings are, however, generalizable and applicable to other groups. Consequently, our goals in this paper are 1) to establish the degree to which the various methods of calculating mechanical energy are correlated, and 2) to investigate whether or not, and if so to what degree, do the prediction methods explain the variation in metabolic energy expenditure.

## Materials and Methods

In order to relate metabolic energy consumption to mechanical energy calculations, eight people (4 men; 4 women) were recruited. Their volumetric rate of oxygen consumption (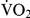) was determined as described in detail below. Although other empirically-determined methods are available (e.g. [16], [19]), the equation endorsed by the American College of Sports Medicine [20] was used as the representative method to predict metabolic energy for walking (ACSM-walk). Mechanical energy was calculated using the methods detailed in Table 1 and included methods that calculate the external, internal and combined work required to move the body. Mechanical energy approaches included those based on sacral motion, ground reaction force profiles, force production assessed via ground contact time, joint moments (via forward dynamics), and an inverse dynamics simulation developed in SimMechanics, a module within Matlab (R2010a, The Math Works, Natick, MA), which is detailed below. Prediction of metabolic energy using dynamic models with muscles modeled as spring elements was not evaluated, because individual variation cannot be modeled at this time.

### Subjects

The premise behind our sampling scheme was to represent within our sample typical variability in adult humans. Consequently, the only exclusion criterion for this study was the presence of inherent gait pathology (e.g. leg length discrepancy) or substantial injury to the lower extremity (e.g. that requiring surgical repair). Table 2 provides subject-specific values used in the analyses described below. Mass was assessed with a standard scale, calibrated and read to the nearest 0.1 kg. Stature was assessed to the nearest cm. Crural index was calculated from the marker positions that are described below and equaled the ratio of calf length (center of lateral knee to lateral malleolus) and thigh length (vertical distance from greater trochanter to center of lateral knee). Note that calculating crural index in this way will yield higher values than the traditional method based on measuring disarticulated bones. Body mass index (BMI) was calculated as body mass divided by the square of stature. The Institutional Review Board, Committee EG of the University of Washington approved this study and all subjects provided written informed consent before participating.

**Table 2:** Subject characteristics.

Subject id	Sex	Age (yr)	stRMR (mlO_2_/s)	Mass (kg)	Stature (m)	Thigh length (m)	Calf length (m)	Foot length (m)	Pelvic width (m)	BMI (kg/m^2^)	Crural index
6	M	22.0	205	52.4	1.60	0.371	0.345	0.104	0.253	20.5	0.92
10	F	53.1	250	70.0	1.52	0.353	0.346	0.098	0.288	30.3	0.98
11	M	25.5	255	59.7	1.70	0.380	0.371	0.116	0.289	20.7	0.98
13	M	23.2	353	84.2	1.85	0.470	0.445	0.127	0.268	24.1	0.95
15	F	32.3	169	62.4	1.68	0.435	0.387	0.124	0.235	22.1	0.89
27	M	26.2	371	84.2	1.75	0.424	0.406	0.129	0.260	26.9	0.96
38	F	44.7	191	76.0	1.72	0.410	0.401	0.112	0.282	25.7	0.98
39	F	24.1	193	55.2	1.56	0.337	0.363	0.111	0.240	22.7	1.08

### Metabolic data collection and analysis

Subjects walked on a level treadmill while their 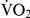 (in mlO_2_/min) was determined by a VO_2000_ oxygen analyzer (Medgraphics, Minneapolis, MN). The subjects wore a neoprene mask that was attached to the oxygen analyzer via a pneumatach and plastic tubing. Breath-by-breath measurements of 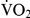 and 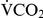 were obtained and all respiratory quotients were <1.0 to ensure that the exercises were conducted in aerobic conditions.

Resting metabolic rate while standing (stRMR) was determined at the beginning of the exercise session. Subjects were then asked to determine their self-selected slow, normal and fast walking velocities and given time to accommodate to the treadmill and the mask prior to data collection. Individual trials at a velocity lasted for 4 min with 4 minutes of standing at rest between trials. Four minutes was chosen because this gave sufficient time to establish a plateau while exercising and allowed 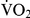 to return to resting values between trials. The order of the trials was randomly determined.

One minute averages of the raw breath-by-breath data were computed. The average from minutes 3 and 4 of each trial were used in subsequent analyses. The difference between the measured 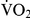 from minutes 3 and 4 and stRMR was calculated and these values were then used to develop a subject-specific, linear equation that predicted 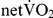 (in mlO2/min) from velocity (all p's <0.001 and r^2^  = 0.68–0.99). This equation was used to determine 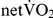 for the appropriate motion analysis trial velocity (described below). The measured velocity of each trial was matched to the individual's velocity-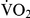 curve and this metabolic value was used in subsequent analyses.

### Motion and force data collection and analysis

Motion and ground reaction force profiles of the subjects were assessed in a human motion analysis laboratory, which is equipped with a 6-camera Qualysis (Gothenburg, Sweden) infrared system to capture 3-dimensional motion and an embedded force plate to measure ground reaction force. Data were collected at 120 Hz and stored for later analyses. Calibration of the motion capture volume occurred immediately before each subject's test in accordance with the manufacturer's instructions and marker positions were determined to be within 2 mm of their known position. A standard marker set was attached to the following landmarks of each subject: left and right acromion, anterior superior iliac spine, greater trochanter, superior patella, lateral knee, tibial tuberosity, lateral malleolus, posterior heel, and the space between the second and third metatarsal heads and the superior sacral border. Subjects wore athletic shoes with socks that exposed the malleoli, spandex shorts, and sleeveless shirts during the walking trials. Sacral, ASIS, and trochanteric markers were placed over the landmarks on the shorts; heel and metatarsal head markers were placed on the shoes. Other markers were attached directly to the skin. Once placed, these markers remained in place for all trials. Additional markers on the left and right medial knee and medial malleolus were used to determine joint widths in an initial standing trial and then removed.

Subjects walked at their self-selected slow, normal and fast velocities 10 times across the force plate (5 trials each with left and right foot contact with the force plate), which resulted in 30 walking trials for each subject. Subjects were instructed to watch a point on the laboratory wall and maintain a smooth motion. Trials in which the appropriate foot did not fully contact the force plate or which had excessive gaps in marker data (which can occur when the arm does not swing enough to reveal the trochanteric marker to the cameras) were redone.

After post-processing using standard Qualysis software, analyses were conducted with custom-developed LabView (National Instruments Corporation, Austin TX) programs to determine 1) peak hip range of motion for each trial to be used in the Matlab simulation described below; 2) external work using the sacral marker motion, ground reaction forces, and force production methods; and 3) total work from joint moments.

### Matlab model

We developed a mechanical model using SimMechanics, the mechanical simulation module of Matlab (R2010a, The Math Works, Natick, MA). The model included rigid bodies representing the left and right thigh, calf and foot segments and a pelvis/trunk which linked the two legs. Motion of the knee and ankle joints was restricted to the para-sagittal plane while that of the hip joints was allowed in all three planes. Two groups of inputs were required by the SimMechanics model: limb segment parameters and angular displacements.

Thigh, calf and foot segmental variables include segment length and proximal and distal circumferences. Segment lengths and joint widths were determined from the initial standing trial of the motion analysis using the distance between appropriate markers. Knee and ankle circumferences were derived assuming that the joint widths were diameters. Thigh circumference was calculated from a linear regression equation relating thigh circumference to knee circumference, developed from the data contained in the 1988 US Army Anthropometric Survey [61]. Mass moments of inertia and segment masses were calculated assuming that the thigh and calf were idealized truncated cones of circular cross-section, while the foot was assumed to be a rectangular block. All segments were assumed to be of homogenous density. These shapes are similar to those recommended by others [62]. For the thigh segment, the thigh circumference was used as the proximal diameter of the truncated cone and the knee circumference as the distal diameter. For the calf segment, the knee circumference was the proximal diameter and the ankle circumference the distal one.

Typical hip angular displacement profiles were obtained for average adult humans walking at slow, normal and fast velocities [63] and have been used previously in a similar model [31]. These general profiles were modified to reflect the maximum hip excursion of each subject for each trial. Knee and ankle angular velocities were not subject-specific.

Linear velocities of the centers of gravity and angular velocity for the segments were output from the SimMechanics model (which used an inverse dynamic solution) and were reported for each limb segment for temporal increments from 0–50% of stride cycle. These were used to calculate internal work at each temporal increment from the standard equations, similar to the procedure of Cavagna and Kaneko [34]. Full (100%) energy transfer was allowed between the thigh, calf and foot of the same limb, but not between a limb and the body, as has been done previously [31], [64]. Intra-limb energy transfers are feasible because the accelerations and masses and, consequently, forces, are similar, but this is not the case between the body and the limbs [64].

The internal energy of a limb at one temporal increment was compared to that of the next temporal increment. If the energy of a later increment was greater than that of the previous increment, extra energy was added to the system to create motion. If a subsequent increment had a lower energy than the previous, this energy was not stored for later use and was set to zero. This approach is similar to previous work [31], [64]. The extra energy required for each interval was summed across the step for left and right legs. Internal work for that iteration was the sum of the extra energy required by the left and right legs. External work was calculated from the change in position of the body's center of gravity.

Every trial for each subject from the motion analysis was evaluated separately using the SimMechanics model.

### Mechanical calculations

The individual data described above were used to calculate mechanical energy using several methods as detailed in Table 1. These approaches can be grouped into those that calculate external work (EXT-xx), internal work (INT-xx) or the combination of internal and external work (COMB-xx). External work was calculated using contact time via force production (EXT-FP), ground reaction forces (EXT-GRF), and sacral motion (measured by a sacral marker (EXT-SAC) and determined by the Matlab model (EXT-MAT)). Internal work was calculated via the Matlab model (INT-MAT). Combined work was determined from joint moments (COMB-JM) and the sum of internal and external work via the Matlab model (COMB-MAT).

### Statistical analysis

The pairwise correlation coefficients between metabolic and mechanical energy approaches were calculated to see if the methods varied together. Then, each method of predicting energy expenditure was regressed against 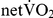 (except for ACSM which was regressed against 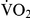) to see whether or not it accurately reflected the change in 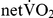 with increasing intensity. Both ordinary least squares (OLS) and maximum likelihood estimation (MLE) statistical techniques were used. As detailed below, OLS analysis allows for many of the relationships to be examined using familiar statistical criteria such as coefficients of determination, but some relationships are more complex and a formulation to evaluate them exists only for MLE. We chose to do both to allow readers who are less familiar with MLE to access the analysis from a familiar perspective. The anthropometric variables listed in Table 2 were also included in a stepwise OLS regression.

Because previous work [65] indicated that considerable between individual variability exists in walking 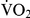 and that at least some of this variability is due to physiological factors for which we are not currently able to control, we wanted to distinguish variability that exists within an individual from that which exists between individuals. This is important because it provides information regarding the degree to which the method accounts for these two sources of variation (within and between individuals). The goal of collecting this kind of information is to begin to discern where the methods are weak and need more sophisticated approaches.

To accomplish this goal, we used hierarchical linear modeling to examine the relationship between our dependent variable, 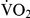 (metabolic energy), and the potential independent variables, i.e., the various approaches to calculating of mechanical energy. This type of statistical procedure provides two advantages over non-hierarchical techniques. First, because each subject was measured during multiple trials, data points within a subject are not independent and clustering is required to account for the non-independence of the repeated measures. Second, measured metabolic energy can be explained by both variance within an individual subject and variance that exists between subjects. Hierarchical linear modeling partitions the variance of the independent variable and determines the separate and combined contributions to predicting the dependent variable.

In the first analysis, subjects were considered clusters of data points (an individual subject measured during multiple trials of walking). In this analysis, all subjects are constrained to have the same slope (termed “fixed slope”) relating measured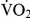 to the predicted metabolic or mechanical energy, but each subject is permitted to have a different intercept (termed “random intercept”). Using OLS, these analyses result in three coefficients of determination: one for the within subject variance, one for the between subject variance, and one using the total combined variance. Coefficients of determination are an absolute measure of the degree to which a statistical model incorporating independent variables explains the variability of the dependent variable. The fixed slope-random intercept analyses were repeated using MLE.

In the second set of analyses, both the intercept and the slope were allowed to vary between subjects (termed random slope-random intercept) using MLE. (This analysis was not done using OLS because the formulation does not exist [66].) The MLE results were interpreted using the Akaike information criterion (AIC) and the Bayesian information criterion (BIC). Information criteria are relative (i.e., they are not equivalent to coefficients of determination) and are used to select among potential models. Models with lower information criteria have a better balance between accuracy of prediction and the number of independent variables included.

All statistical analyses were accomplished in StataSE (Version 9, Stata Corporation, College Station, TX). Where appropriate, statistical significance was set at an alpha of p ≤ 0.05.

## Results

All methods of predicting 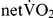 were correlated with each other (Table 3), with correlation coefficients ranging from 0.42–0.99.

**Table 3:** Correlation coefficients of metabolic and mechanical energy approaches.

Variable name	ACSM	EXT-FP	EXT-GRF	EXT-MAT	EXT-SAC	INT-MAT	COMB-JM	COMB-MAT
ACSM	1							
EXT-FP	0.91	1						
EXT-GRF	0.90	0.83	1					
EXT-MAT	0.90	0.87	0.94	1				
EXT-SAC	0.69	0.55	0.42	0.47	1			
INT-MAT	0.90	0.87	0.89	0.96	0.48	1		
COMB-JM	0.70	0.70	0.52	0.56	0.71	0.62	1	
COMB-MAT	0.90	0.87	0.93	0.99	0.47	0.97	0.57	1

Using OLS and requiring a fixed slope for all subjects but allowing a random intercept for each (fixed slope-random intercept), the coefficients of determination were calculated for all predictive methods relative to 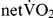 (Table 4). All methods predict 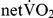, explaining 38–76% of the overall variation. The ACSM method, which is the representative metabolic energy approach, explains more of the variation in 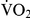 than do the mechanical energy methods. Among the mechanical energy methods, the method using sacral motion was the poorest predictor, explaining 38% of the variation in 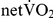. All methods were good at predicting within subject variation, but only the ACSM, force production, and joint moment methods were able to account for between subject variation.

**Table 4:** Coefficients of determination (r^2^) between energy prediction methods and net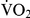, including within a subject, between subjects and overall effects using OLS and requiring fixed slopes but allowing random intercepts.

	net 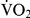			net 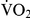 and mass or crural index**		
Variable name	Within a subject r^2^	Between subjects r^2^	Overall r^2^	Within a subject r^2^	Between subjects r^2^	Overall r^2^
ACSM*	0.91	0.74	0.76	0.91	0.76	0.79
EXT-FP	0.83	0.85	0.73	0.83	0.85	0.83
EXT-GRF	0.82	0.14	0.45	0.82	0.46	0.61
EXT-MAT	0.88	0.14	0.48	0.88	0.65	0.72
EXT-SAC	0.67	0.31	0.38	0.67	0.55	0.52
INT-MAT	0.86	0.17	0.45	0.86	0.61	0.68
COMB-JM	0.50	0.63	0.55	0.50	0.86	0.68
COMB-MAT	0.87	0.17	0.46	0.86	0.62	0.69

*ACSM regression statistics are for gross 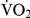.

**ACSM and EXT-GRF are crural index; all other variables methods are mass.

To try to understand why the predictions are less effective at accounting for differences between people than they are within a person, we used our subject-specific variables (Table 2) in a stepwise regression to determine a best-fit equation via OLS assuming a fixed slope-random intercept for each method. The predictive ability of all methods improved with the addition of either crural index (ACSM and ground reaction force methods) or mass (all other methods). Overall predictive ability improved to 52–83% with the addition of a subject specific variable (Table 4).

We further tested the efficacy of the predictive methods by employing MLE. We repeated the fixed slope-random intercept analysis that was completed using OLS and added another analysis, where we allowed the intercept and the slope to vary among subjects (random slope-random intercept). As with the OLS results, the MLE results (Table 5) indicate that all methods produce predictions of similar usefulness and all methods, except the ACSM equation, exhibit better fits with random slope-random intercept than fixed slope-random intercept. The Matlab model and the force production methods produce the best results, while the joint moments and sacral motion the worst.

**Table 5:** Information criteria for MLE for three sets of assumptions: fixed slope-fixed intercept, fixed slope-random intercept, and random slope-random intercept.

		Fixed slope-fixed intercept		Fixed slope-random intercept		Random slope-random intercept	
Method	Variable name	AIC	BIC	AIC	BIC	AIC	BIC
ACSM-walk*	ACSM	2601	2608	2268	2281	**	**
Force production	EXT-FP	2642	2648	2255	2268	1959	1978
CoM-GRF	EXT-GRF	2650	2657	2321	2335	2089	2108
CoM-sacrum-model	EXT-MAT	2664	2671	2292	2304	2018	2038
CoM- sacrum-measured	EXT-SAC	2771	2778	2546	2559	2508	2527
Internal work	INT-MAT	2656	2663	2256	2269	1965	1985
Joint moments	COMB-JM	2708	2714	2603	2616	2554	2574
Model (int + ext work)	COMB-MAT	2662	2669	2283	2296	2002	2022

*ACSM regression statistics are for gross 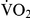.

**random slope-random intercept model not different from fixed slope-random intercept model.

## Discussion

We had two goals at the onset of this research. The first was to establish the degree to which the various methods of calculating mechanical energy are related. The second was to characterize the relationship of the predictive methods with measured energy expenditure.

### Relating mechanical energy approaches

We found that all mechanical energy approaches produced predictions that are significantly correlated. The sacral motion method consistently produces the lowest correlation coefficients, although the relationship between joint moment method and the Matlab simulations are also relatively low (but still >0.5). We anticipated that the methods would be correlated, because all approaches are different mathematical descriptions of the same biological phenomenon.

Of the four ways to calculate external work (i.e., the mechanical energy required to move the whole body), all are correlated, but the method that uses measured sacral motion stands out as having lower correlation coefficients (0.42-0.55) with the others than the others have with themselves (0.83-0.94). The differences in correlation between the prediction methods reflect the difficulty in obtaining the inputs for the calculations. As mentioned previously, all of the methods require assumptions about inputs needed to calculate energy. The variables of body mass, segment lengths, and velocity are relatively simple to measure accurately. The values that are used for the movement of an anatomical landmark, however, have error associated with measuring the marker's position (that can be assessed rigorously and is less than 2 mm) plus error from any movement that occurs between the marker and the landmark (that is of unknown magnitude). For instance, we believe that the cause of the sacral motion discrepancy is that the sacral marker must be placed on the subject's clothing that, even though tightly fitting spandex, does not adhere to the skin as closely in this area as it does at ASIS or greater trochanter. Consequently, our sacral markers may have tended to vibrate or wiggle more than do more closely adhering markers. This observation is based solely on visual assessment and we present it only as a suggested explanation for the discrepancy. Nonetheless, movement in location can become a spike when position is differentiated with respect to time to obtain velocity. Even though smoothing techniques are used, velocity spikes cause anomalous spikes in the energy expenditure calculation. Measuring sacral motion with a marker attached to shorts, then, seems to be the least attractive method to calculate external work.

Some variables, like mass moment of inertia, are impossible to measure in living creatures and have to be estimated. In our case, we assumed that the thigh and calf could be idealized as truncated cones of homogenous density and circular cross-section. We further assumed that the thigh circumference could be estimated from knee circumference. Inaccuracies in the estimation of segmental inertias would affect the Matlab model, but it does not appear to be substantially different from the other methods.

### Characterizing the relationship between predicted and measured energy expenditure

The second of our goals was to characterize the relationship of the predictive methods with measured energy expenditure. We did this using both OLS and MLE statistical techniques. Using OLS, as expected, the ACSM method predicts 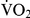 well, explaining 76% of the overall variation in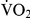, while the force production method explains 73% of the overall variation in 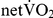. Both of these methods rely on body mass and a measure of intensity (in the form of velocity or ground contact time) to determine energy expenditure.

Mass and intensity variables have been shown to be particularly relevant to locomotor energy expenditure both using metabolic and mechanical energy approaches in all species studied [24]. It seems, therefore, fundamental at this point that any method to predict energy expenditure must incorporate the effect of these two quantities. Body mass and intensity do not, however, explain all the variation in energy expenditure and any possible effects of shape differences within the body cannot be explored by using only these two variables. The other predictive methods, however, produced lower coefficients of determination than either ACSM or force production methods, but this overall lower explanatory ability was due to particularly low between subjects predictive ability. The within subjects r^2^ for all the other methods (except sacral motion and joint moments) was similar to that of ACSM and force production.

To explore this further, we included the subject-specific anthropomorphic variables in a stepwise OLS regression with a fixed slope and random intercept to see if one of the variables would improve the between subjects predictive ability. Although adding a subject-specific variable improved the between subjects fit for all methods, the improvement was marginal for either the ACSM or joint moment method. The other methods saw more improvement. Mass and crural index emerged as the most significant additional predictor and both were independently and positively predictive of measured 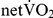
in the presence of each other and the predicted metabolic or mechanical energy. We did not, however, have sufficient participants to include more than one subject-specific variable in the OLS regressions. Given that mass is already accounted in all predictive methods, it seems likely that it is acting as a proxy for another subject-specific variable, perhaps a physiological one, that we did not assess. We could speculate that a likely candidate for this is some measure of “physical fitness,” but we have no data to address the subject. Another possibility is that body mass is acting as a proxy for activated muscle volume [44] or the muscular energy used in joint stabilization. Dynamic models that incorporate muscles and allow these muscles to drive the simulation [32], [41], [42], [43] are one way to test this possibility and we look forward to the further development of the methods and the opportunity to test this possibility with them.

It is also unclear how crural index is functioning. Other work has indicated that calf length is negatively associated with energy expenditure [67], but whether or not it is functioning as a causal or proxy agent is unclear. It is also possible that crural index (or body mass) is acting as a proxy for effective limb length (the distance between the hip and the center of rotation of the hip during stance) [68], but it is unclear why a length variable would not have been a more predictive proxy. More data is needed to explore these relationships.

Using MLE, we repeated the fixed slope-random intercept analysis which we completed using OLS and were able to extend the analysis to a random slope-random intercept statistical model. All mechanical energy approaches benefited from inclusion of a random slope. Using either the Akaike or Bayesian information criteria, the predictive methods, with the exception of measured sacral motion, have similar likelihoods of representing the measured data appropriately. The MLE regressions, therefore, mirror those obtained using OLS.

Within a subject, the metabolic and mechanical methods produce excellent agreement with measured 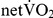, but between subjects the methods are less effective. We propose that the explanatory ability of a method within an individual reflects directly on those factors that vary among trials (e.g. velocity, hip angular excursion), while that among individuals reflects the factors that vary among trials *and* among individuals (e.g. velocity, anthropometrics, physiological factors). Consequently, in the within subject statistics, which are most-easily appreciated by examination of the coefficients of determination shown in Table 4, each person is compared only to themselves. Thus, the fit of that part of the statistical model is due to the amalgamation of the predictive ability of the method within a person across all people. This approach has been useful in evaluating growth curves where similar patterns are present among individuals in the presence of substantial intra-individual variation (Rabe-Hesketh and Skrondal 2005).

### Summary

The general picture that emerges from these analyses is that all mechanical approaches are predictive of 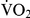 with approximately the same explanatory ability and they are all correlated with each other. This result was expected. Although within subject variation (i.e., the variation introduced solely by intensity) was well-matched, the more unexpected result here is that variation among individuals was not particularly well-predicted with any method except joint moments, which was less able to replicate intra-individual variation.

The chief limitation of this work is the small sample size, especially given that it includes women and men with different physical activity levels and of different ages. The reason to sample broadly was to be able to generalize, but that choice may have limited our ability to detect subtle differences. Nonetheless, we believe that the results of our analyses demonstrate that the choice of methodology should be dependent on the question(s) being asked and not on any perceived theoretical superiority of the method. Inherent in this analysis is our assumption that the principles of Newtonian mechanics and the methods that flow from it are important to the calculation of locomotor energy expenditure and are sensitive to variability in the variables of both large (interspecific) and small scale (intraspecific) analyses. Body mass and velocity are significant predictors of metabolic function in empirical studies of humans (e.g. [16], [19], [20]) and of diverse groups of animal (e.g. [24]). These same variables are also critical components in the mechanical formulations, suggesting causality to us. When the value of body mass and/or velocity span orders of magnitude, their effect may well overwhelm any other more subtle effects (e.g. kinematic differences or segment inertias), but within species, body mass and velocity vary much less and subtle effects may contribute more to the observed variation in energy expenditure. While the major drivers of energy expenditure (mass and intensity) are accounted in all the methods, more subtle potential contributors can only be addressed by methods that incorporate their effect.

In a general example, changes in shape, like variations in crural index, can be addressed by the forward and inverse dynamics methods, because they incorporate a linked-segment model which has individual limb segment lengths, but not by the force production method. This is not to say that there is anything wrong with the force production method, just that it does not include the level of detail that is needed to explore the specific question of the influence of crural index on energy expenditure.

Specific examples can be easily found in the current anthropological literature. On the one hand, Jungers and colleagues [69] suggest that kinematic differences from modern humans existed in the gait of *Homo floresiensis,* because the foot of *H. floresiensis* is proportionately longer than that of modern humans. This long foot requires that the knee be more flexed at mid-swing, so that the toes can clear the ground. This change in angles potentially changes energy expenditure, but it can only be evaluated with the inverse dynamics approach and not the other mechanical methods, because only inverse dynamics addresses kinematic variability. In this case, a mechanical model is the only choice for the analysis. A metabolic approach for this problem is less-easily implemented or interpreted. One could artificially increase the foot length in an experimental group of humans (by, for instance, requiring them to wear shoes with exaggerated toe boxes), but it is difficult to know if any difference in energetics between the long-foot and normal-foot groups that might be found is due to foot length or to the inexperience of the subjects in dealing with long feet.

On the other hand, determining the energetic expenditure of pursuit hunting in *Homo* [70] would probably be best done with a metabolic approach using empirically-determined equations. The principle reason is that modern humans are available for study so little extrapolation is required. Human data can be directly used to evaluate the question of interest.

Although we have drawn our examples here from the locomotion of modern humans and their extinct ancestors and relatives, the ideas are applicable to other groups as well. The ability to choose analysis method based on the type of information available should be useful in general to biologists, particularly given that extant nonhuman species are often more difficult to study than humans. Although we acknowledge that this work should be extended to other species, we are encouraged that the methods, when applied to humans, produce consistent results.

### Conclusion

Our critical points are that the choice of method is dependent on the question of interest and that no method is intrinsically better or worse than another. The study of locomotion, whether in creatures from the deep past or modern people, benefits from multiple lines of inquiry from diverse perspectives. The performance of any simulation of reality is dependent on the quality of the data available as inputs, the assumptions made to allow the calculations to proceed, and the question of interest.
